# Supplementary material for: Melanoma antigens in pediatric medulloblastoma contribute to tumor heterogeneity and species-specificity of group 3 tumors
Source: Acta Neuropathol Commun. 2025 Jul 28;13:164. doi: 10.1186/s40478-025-02055-3 (PMC12302604; doi:10.1186/s40478-025-02055-3)
Supplement: Supplementary file 2 — Additional file2 [file 40478_2025_2055_MOESM2_ESM.pptx]

## Slide 1
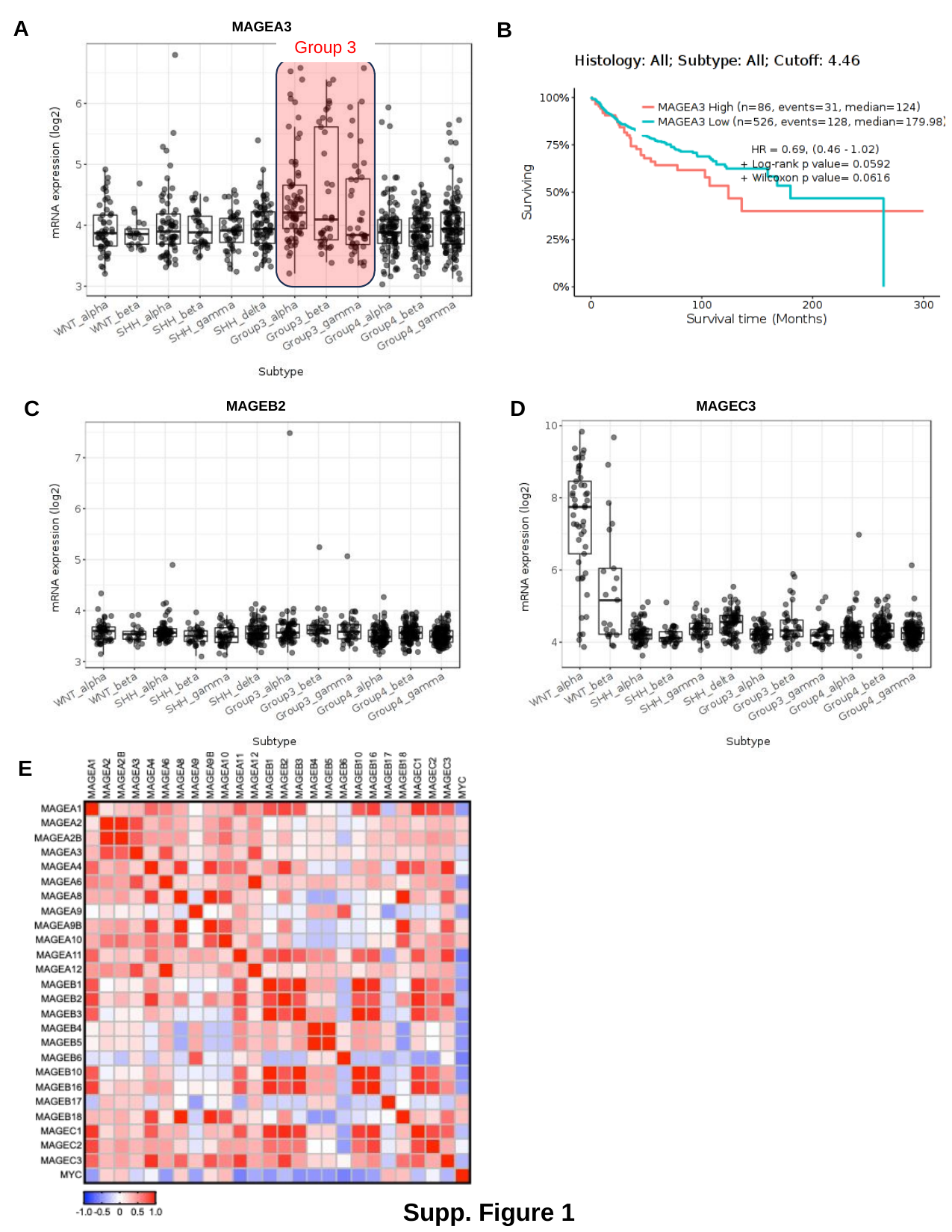

A
B
MAGEA3
Group 3
C
D
MAGEB2
MAGEC3
E
Supp. Figure 1

## Slide 2
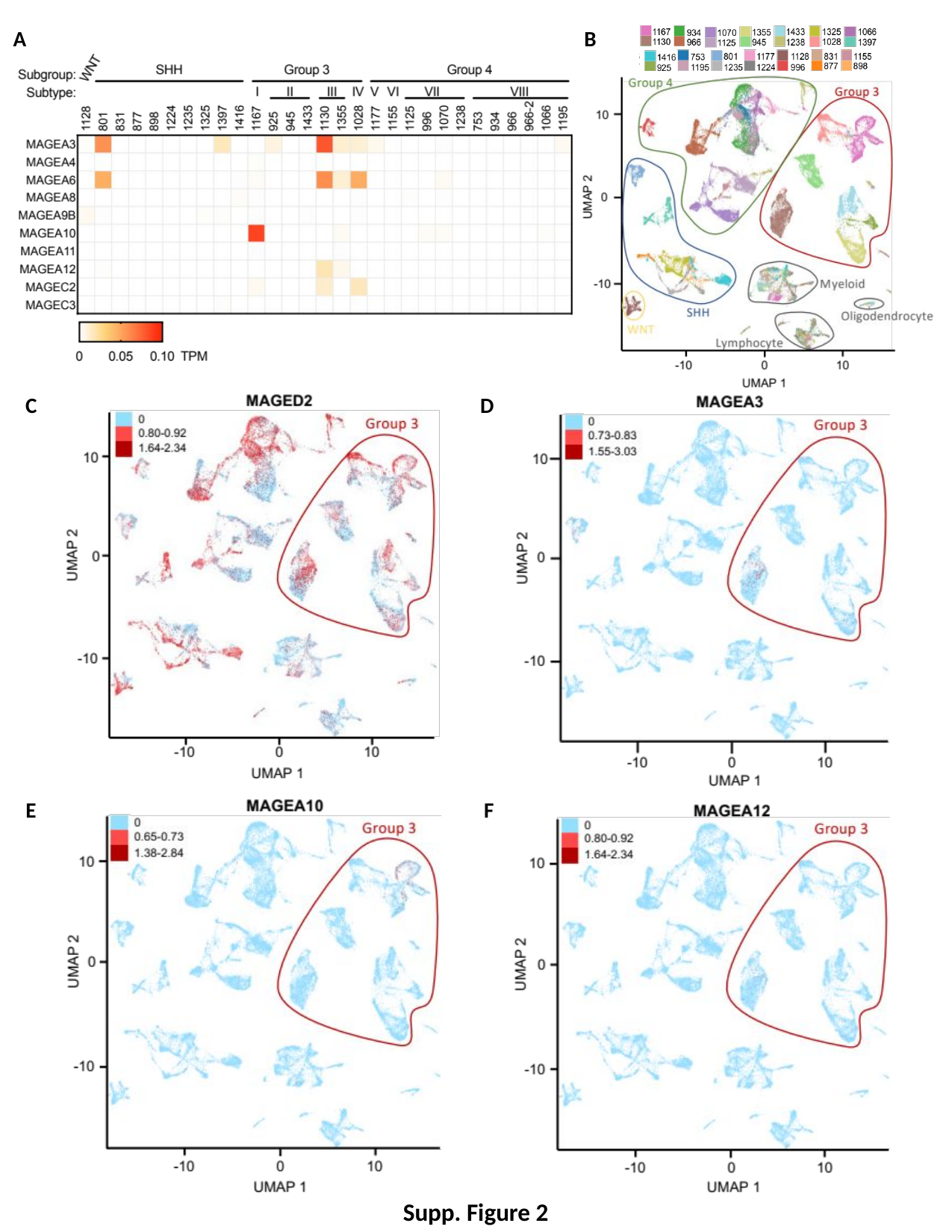

A
B
C
D
E
F
Supp. Figure 2

## Slide 3
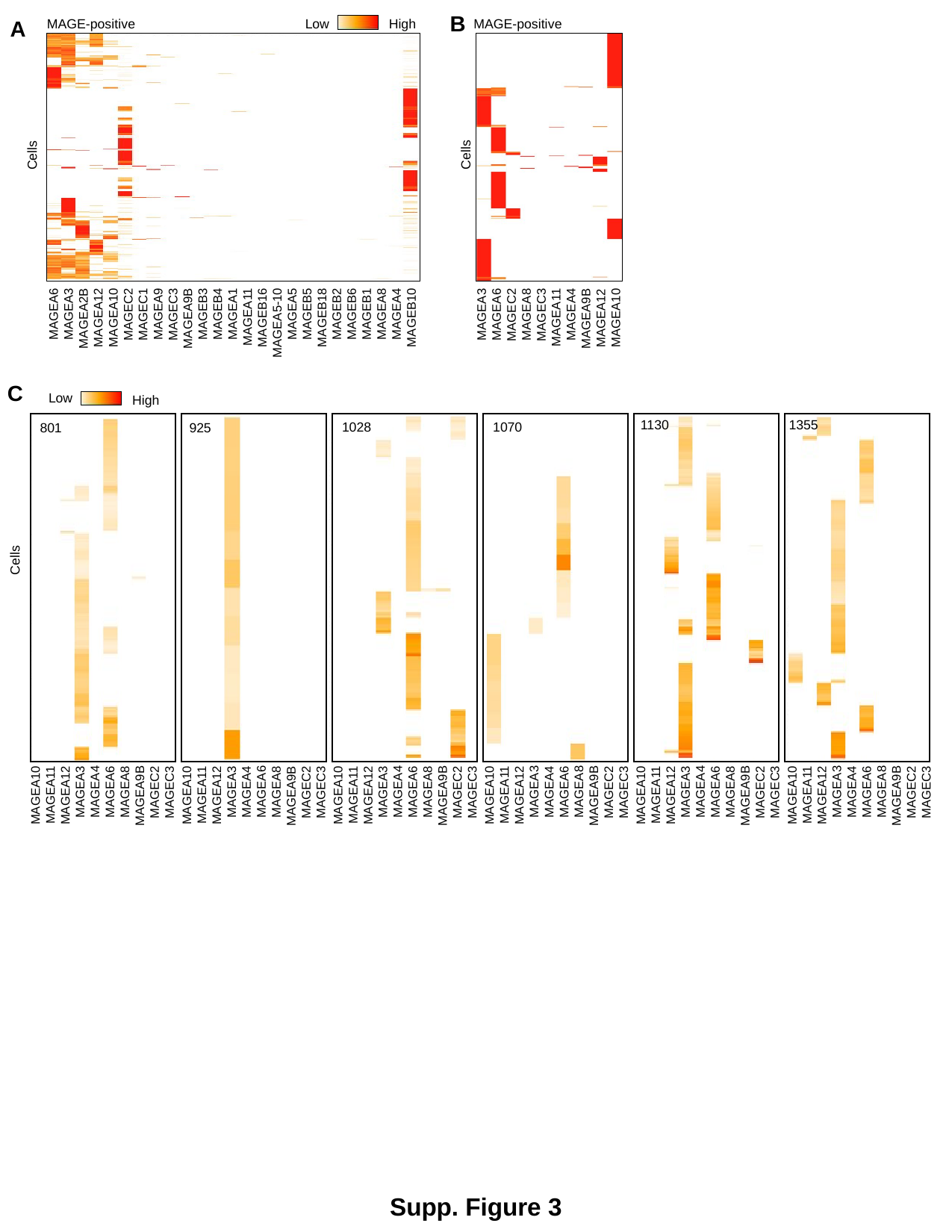

B
A
MAGE-positive
Low
High
MAGE-positive
MAGEA6
MAGEA3
MAGEA2B
MAGEA12
MAGEA10
MAGEC2
MAGEC1
MAGEA9
MAGEC3
MAGEA9B
MAGEB3
MAGEB4
MAGEA1
MAGEA11
MAGEB16
MAGEA5-10
MAGEA5
MAGEB5
MAGEB18
MAGEB2
MAGEB6
MAGEB1
MAGEA8
MAGEA4
MAGEB10
Cells
Cells
MAGEA3
MAGEA6
MAGEC2
MAGEA8
MAGEC3
MAGEA11
MAGEA4
MAGEA9B
MAGEA12
MAGEA10
C
Low
High
1130
1355
1028
1070
801
925
Cells
MAGEA10
MAGEA11
MAGEA12
MAGEA3
MAGEA4
MAGEA6
MAGEA8
MAGEA9B
MAGEC2
MAGEC3
MAGEA10
MAGEA11
MAGEA12
MAGEA3
MAGEA4
MAGEA6
MAGEA8
MAGEA9B
MAGEC2
MAGEC3
MAGEA10
MAGEA11
MAGEA12
MAGEA3
MAGEA4
MAGEA6
MAGEA8
MAGEA9B
MAGEC2
MAGEC3
MAGEA10
MAGEA11
MAGEA12
MAGEA3
MAGEA4
MAGEA6
MAGEA8
MAGEA9B
MAGEC2
MAGEC3
MAGEA10
MAGEA11
MAGEA12
MAGEA3
MAGEA4
MAGEA6
MAGEA8
MAGEA9B
MAGEC2
MAGEC3
MAGEA10
MAGEA11
MAGEA12
MAGEA3
MAGEA4
MAGEA6
MAGEA8
MAGEA9B
MAGEC2
MAGEC3
Supp. Figure 3

## Slide 4
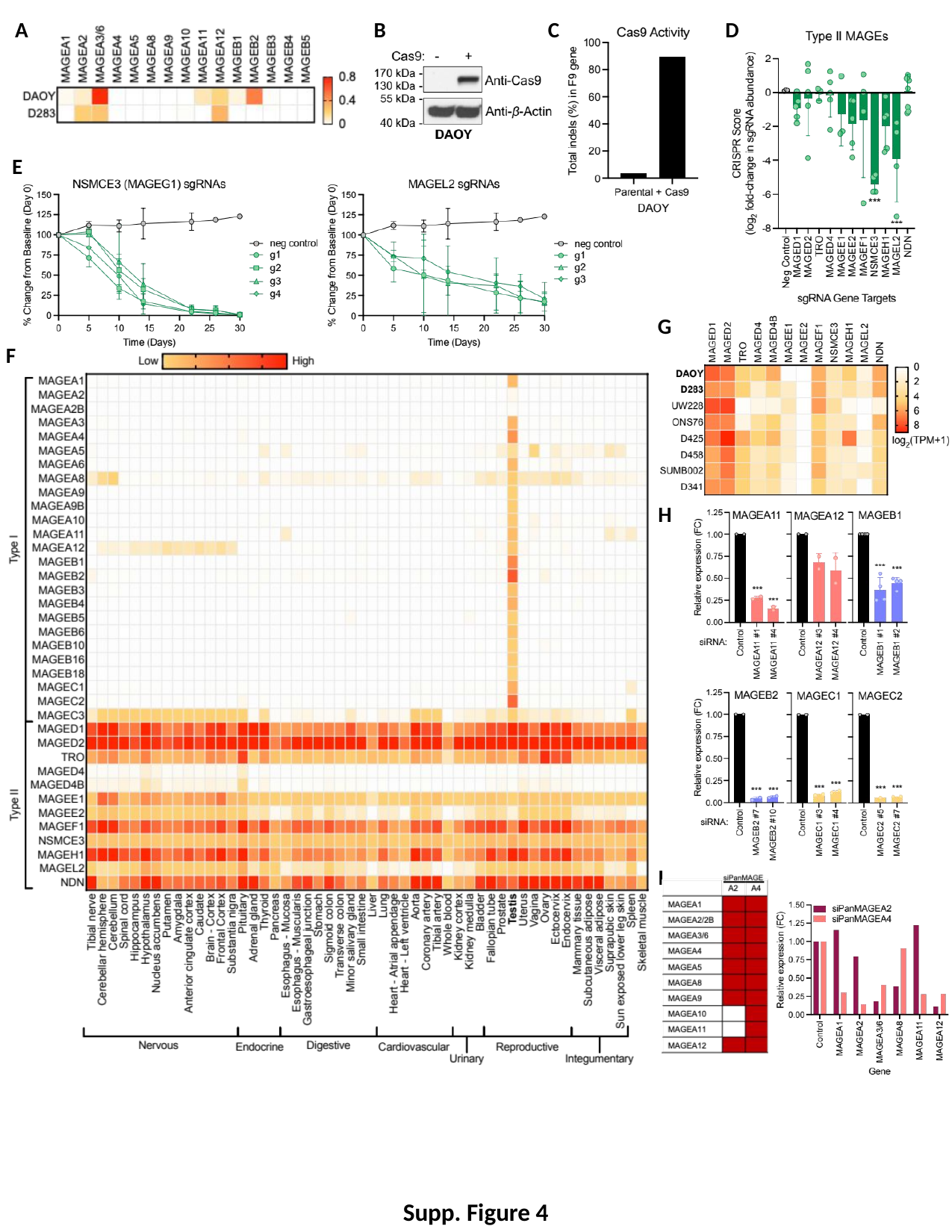

A
B
C
D
E
G
F
H
I
Supp. Figure 4

## Slide 5
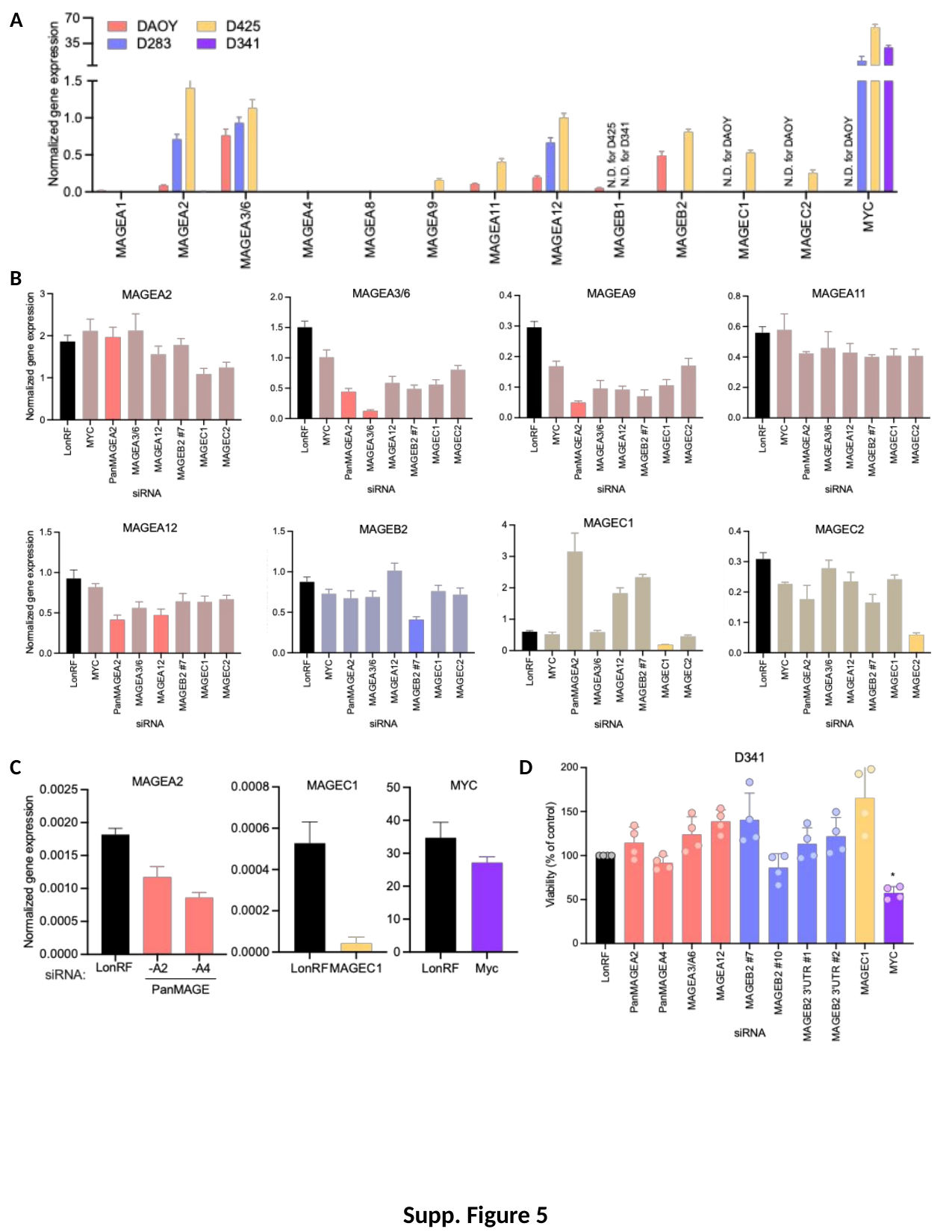

A
B
C
D
Supp. Figure 5

## Slide 6
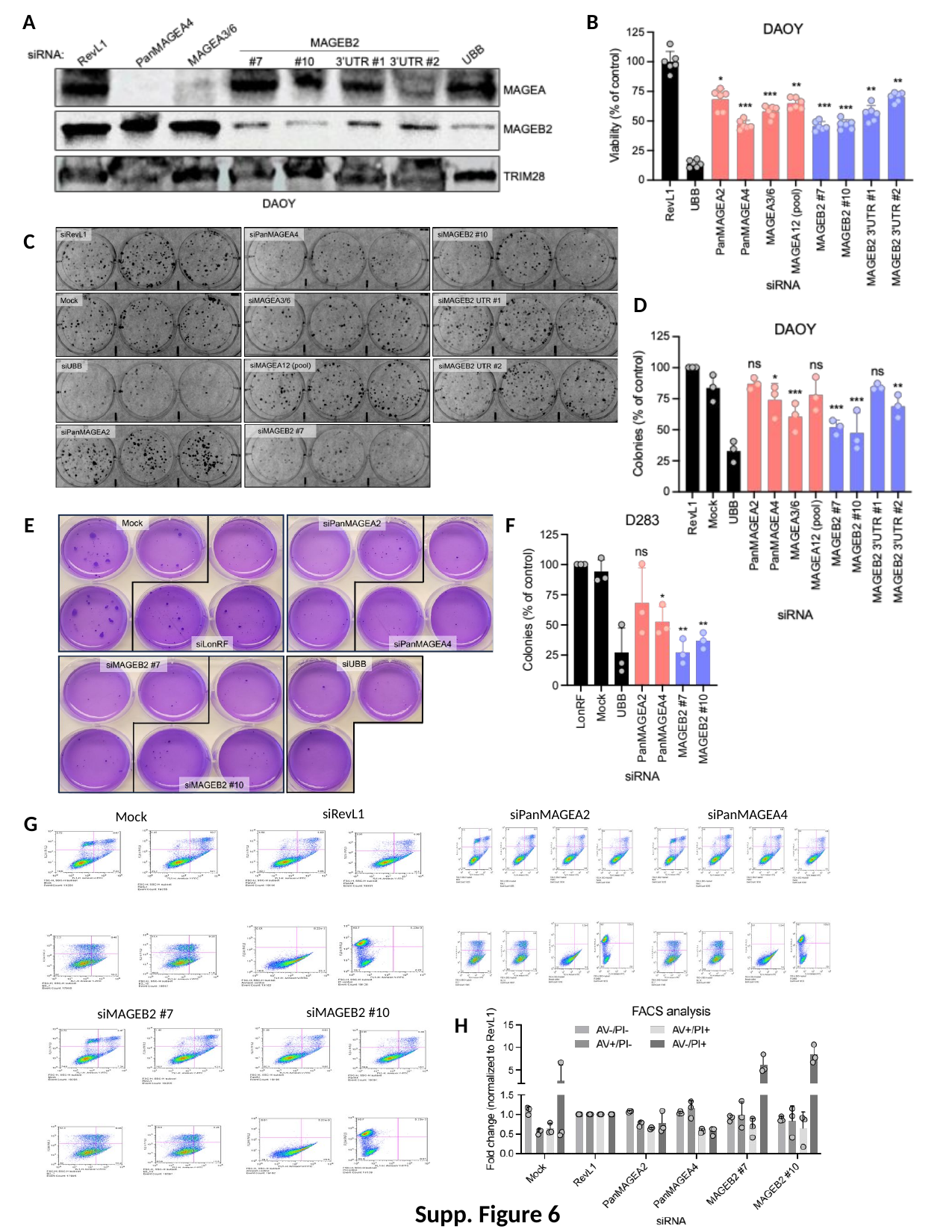

A
B
C
D
E
F
siRevL1
Mock
siMAGEB2 #10
siMAGEB2 #7
siPanMAGEA2
siPanMAGEA4
G
H
Supp. Figure 6
