# Supplementary material for: Melanoma antigens in pediatric medulloblastoma contribute to tumor heterogeneity and species-specificity of group 3 tumors
Source: Acta Neuropathol Commun. 2025 Jul 28;13:164. doi: 10.1186/s40478-025-02055-3 (PMC12302604; doi:10.1186/s40478-025-02055-3)
Supplement: Supplementary file 6 — Additional file6 [file 40478_2025_2055_MOESM6_ESM.pptx]

## Slide 1
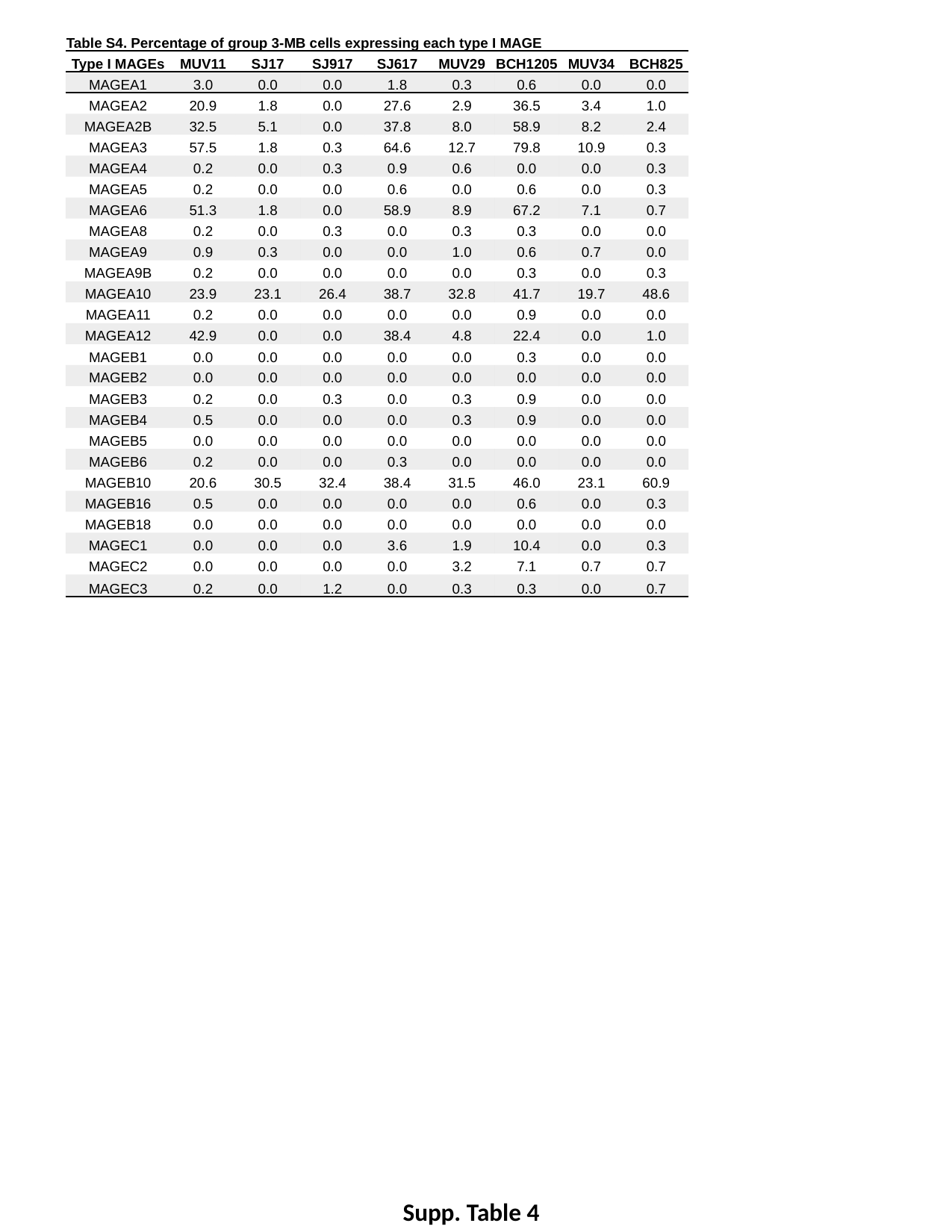

| Table S4. Percentage of group 3-MB cells expressing each type I MAGE | % of cells expressing each (type I) MAGE | | | | | | | |
| --- | --- | --- | --- | --- | --- | --- | --- | --- |
| Type I MAGEs | MUV11 | SJ17 | SJ917 | SJ617 | MUV29 | BCH1205 | MUV34 | BCH825 |
| MAGEA1 | 3.0 | 0.0 | 0.0 | 1.8 | 0.3 | 0.6 | 0.0 | 0.0 |
| MAGEA2 | 20.9 | 1.8 | 0.0 | 27.6 | 2.9 | 36.5 | 3.4 | 1.0 |
| MAGEA2B | 32.5 | 5.1 | 0.0 | 37.8 | 8.0 | 58.9 | 8.2 | 2.4 |
| MAGEA3 | 57.5 | 1.8 | 0.3 | 64.6 | 12.7 | 79.8 | 10.9 | 0.3 |
| MAGEA4 | 0.2 | 0.0 | 0.3 | 0.9 | 0.6 | 0.0 | 0.0 | 0.3 |
| MAGEA5 | 0.2 | 0.0 | 0.0 | 0.6 | 0.0 | 0.6 | 0.0 | 0.3 |
| MAGEA6 | 51.3 | 1.8 | 0.0 | 58.9 | 8.9 | 67.2 | 7.1 | 0.7 |
| MAGEA8 | 0.2 | 0.0 | 0.3 | 0.0 | 0.3 | 0.3 | 0.0 | 0.0 |
| MAGEA9 | 0.9 | 0.3 | 0.0 | 0.0 | 1.0 | 0.6 | 0.7 | 0.0 |
| MAGEA9B | 0.2 | 0.0 | 0.0 | 0.0 | 0.0 | 0.3 | 0.0 | 0.3 |
| MAGEA10 | 23.9 | 23.1 | 26.4 | 38.7 | 32.8 | 41.7 | 19.7 | 48.6 |
| MAGEA11 | 0.2 | 0.0 | 0.0 | 0.0 | 0.0 | 0.9 | 0.0 | 0.0 |
| MAGEA12 | 42.9 | 0.0 | 0.0 | 38.4 | 4.8 | 22.4 | 0.0 | 1.0 |
| MAGEB1 | 0.0 | 0.0 | 0.0 | 0.0 | 0.0 | 0.3 | 0.0 | 0.0 |
| MAGEB2 | 0.0 | 0.0 | 0.0 | 0.0 | 0.0 | 0.0 | 0.0 | 0.0 |
| MAGEB3 | 0.2 | 0.0 | 0.3 | 0.0 | 0.3 | 0.9 | 0.0 | 0.0 |
| MAGEB4 | 0.5 | 0.0 | 0.0 | 0.0 | 0.3 | 0.9 | 0.0 | 0.0 |
| MAGEB5 | 0.0 | 0.0 | 0.0 | 0.0 | 0.0 | 0.0 | 0.0 | 0.0 |
| MAGEB6 | 0.2 | 0.0 | 0.0 | 0.3 | 0.0 | 0.0 | 0.0 | 0.0 |
| MAGEB10 | 20.6 | 30.5 | 32.4 | 38.4 | 31.5 | 46.0 | 23.1 | 60.9 |
| MAGEB16 | 0.5 | 0.0 | 0.0 | 0.0 | 0.0 | 0.6 | 0.0 | 0.3 |
| MAGEB18 | 0.0 | 0.0 | 0.0 | 0.0 | 0.0 | 0.0 | 0.0 | 0.0 |
| MAGEC1 | 0.0 | 0.0 | 0.0 | 3.6 | 1.9 | 10.4 | 0.0 | 0.3 |
| MAGEC2 | 0.0 | 0.0 | 0.0 | 0.0 | 3.2 | 7.1 | 0.7 | 0.7 |
| MAGEC3 | 0.2 | 0.0 | 1.2 | 0.0 | 0.3 | 0.3 | 0.0 | 0.7 |
Supp. Table 4
