# Supplementary material for: Melanoma antigens in pediatric medulloblastoma contribute to tumor heterogeneity and species-specificity of group 3 tumors
Source: Acta Neuropathol Commun. 2025 Jul 28;13:164. doi: 10.1186/s40478-025-02055-3 (PMC12302604; doi:10.1186/s40478-025-02055-3)
Supplement: Supplementary file 7 — Additional file7 [file 40478_2025_2055_MOESM7_ESM.pptx]

## Slide 1
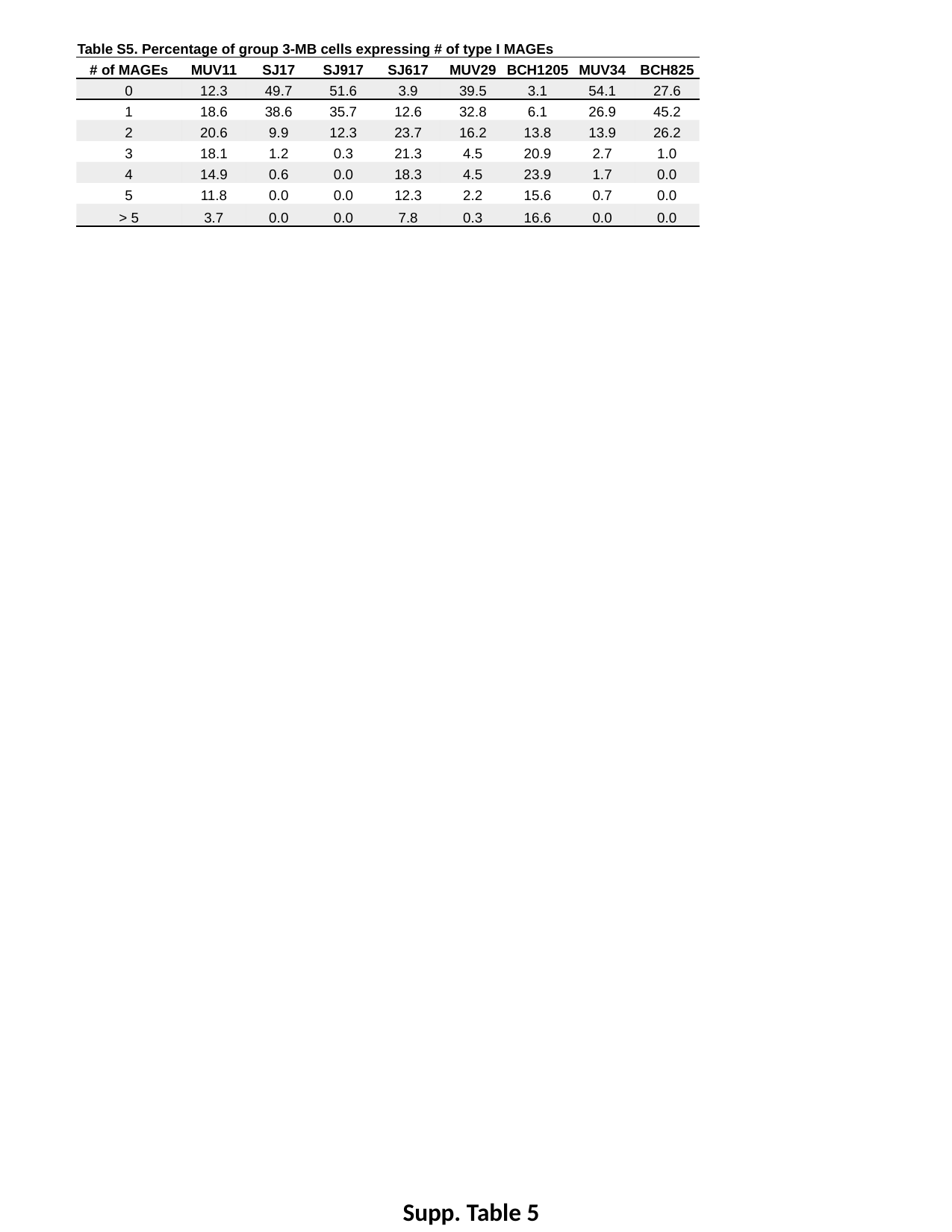

| Table S5. Percentage of group 3-MB cells expressing # of type I MAGEs | % of cells expressing (number) of type I MAGEs | | | | | | | |
| --- | --- | --- | --- | --- | --- | --- | --- | --- |
| # of MAGEs | MUV11 | SJ17 | SJ917 | SJ617 | MUV29 | BCH1205 | MUV34 | BCH825 |
| 0 | 12.3 | 49.7 | 51.6 | 3.9 | 39.5 | 3.1 | 54.1 | 27.6 |
| 1 | 18.6 | 38.6 | 35.7 | 12.6 | 32.8 | 6.1 | 26.9 | 45.2 |
| 2 | 20.6 | 9.9 | 12.3 | 23.7 | 16.2 | 13.8 | 13.9 | 26.2 |
| 3 | 18.1 | 1.2 | 0.3 | 21.3 | 4.5 | 20.9 | 2.7 | 1.0 |
| 4 | 14.9 | 0.6 | 0.0 | 18.3 | 4.5 | 23.9 | 1.7 | 0.0 |
| 5 | 11.8 | 0.0 | 0.0 | 12.3 | 2.2 | 15.6 | 0.7 | 0.0 |
| > 5 | 3.7 | 0.0 | 0.0 | 7.8 | 0.3 | 16.6 | 0.0 | 0.0 |
Supp. Table 5
